# Gut Bacterial Community Determines the Therapeutic Effect of Ginsenoside on Canine Inflammatory Bowel Disease by Modulating the Colonic Mucosal Barrier

**DOI:** 10.3390/microorganisms11112616

**Published:** 2023-10-24

**Authors:** Aipeng Mao, Weigang Zhao, Yuhang Zhu, Fantao Kong, Danyang Chen, Huazhe Si, Chao Xu

**Affiliations:** 1 Institute of Special Animal and Plant Sciences, Chinese Academy of Agricultural Sciences, Research Center for Microbial Feed Engineering of Special Animals in Jilin Province, Innovation Center for Feeding and Utilization of Special Animals in Jilin Province, Changchun 130112, China; mao443418199@163.com (A.M.); zwg1163@126.com (W.Z.); kongfantao@caas.cn (F.K.); chendanyang@caas.cn (D.C.); 2 College of Animal Science and Technology, Jilin Agricultural University, Changchun 130118, China; yuhangzhu199512@163.com

**Keywords:** ginsenoside, inflammatory bowel disease, tight junction protein, adherens junction protein, mucosal bacterial community

## Abstract

Inflammatory bowel disease (IBD) comprises systemic inflammatory conditions primarily affecting the gastrointestinal tract, including Crohn’s disease and ulcerative colitis. This research aims to analyze the clinical symptoms and pathogenesis of a Dextran sodium sulfate (DSS)-induced canine IBD model and evaluate the restorative effect of ginsenoside from a pathogenesis perspective. We established the DSS-induced canine IBD model and studied the pathological mechanisms. Additionally, we examined the therapeutic effect of ginsenosides by assessing the Canine Inflammatory Bowel Disease Activity Index (CIBDAI), C-reactive protein (CRP) levels, colonic tissue morphology, protein expression, and mucosal bacterial community analysis. Our findings revealed a total ginsenoside content of 22.7% in the ginsenoside extract. Animal experiments demonstrated that dogs with IBD exhibited decreased mental state, significantly increased CIBDAI and CRP levels, disrupted colonic epithelial tissue structure, decreased expression of mucin, tight junctions, and adherens junctions, as well as reduced diversity of the colonic mucosal bacterial community. Furthermore, correlation analysis highlighted a total of 38 bacterial strains correlated with physiological indices. Significantly, ginsenoside treatment could improve these symptoms and reverse the relative abundance of some bacterial communities. In conclusion, alterations in the properties of the colonic mucus layer or the reduction in MUC2, its core component, in dogs with IBD can lead to bacterial penetration of the mucus layer and subsequent contact with intestinal epithelial cells, resulting in inflammation. Remarkably, ginsenoside intervention showcased the capacity to positively influence the relative abundance of bacteria and impact the colonic mucus layer properties, thereby offering promising prospects for IBD management and recovery.

## 1. Introduction

In recent years, there has been a significant increase in the incidence and disease burden of inflammatory bowel disease (IBD) globally [1,2,3]. The intestinal epithelium plays a crucial role in maintaining a selectively permeable barrier that facilitates nutrient absorption and waste excretion while preventing the intrusion of luminal contents [4,5,6]. Disruption of the intestinal epithelial barrier integrity can result in excessive leakage of bacterial antigens into the mucosa, triggering inflammatory reactions and progressive damage to the intestinal epithelial cells. This, in turn, leads to further antigen leakage and aggravates inflammation, compromising barrier integrity even more [7,8]. The epithelium maintains its selective barrier function through the intricate and dynamic system of protein–protein interactions that connect adjacent cells and seal the intercellular space [9]. This protein network is composed of three adhesion complexes: tight junctions, adherens junctions, and desmosomes. Tight junction proteins, such as Occludin, Claudins, and Zonula, are located at the apical region of the epithelial intracellular junctions [10], regulating ion and solute flux as the “gate function” and maintaining cell polarity as the “fence function” [11]. Adherens junctions and desmosomes contribute to adhesive and mechanical properties vital for barrier function, although they do not fully seal the paracellular space.

The immune system in the large intestine is protected from commensal microorganisms and rare intestinal pathogens by a single layer of diverse epithelial cells, which are covered by a dense and adherent inner mucus layer and a looser outer mucus layer [12]. The integrity and functionality of the inner mucus layer are crucial in preventing bacterial penetration and subsequent colonic inflammation [13,14,15]. Several factors have been identified to contribute to changes in mucus properties. For instance, deficiencies in TRIM34 can lead to reduced secretion of MUC2 by goblet cells, resulting in defects in the inner mucus layer [16]. Additionally, NLRP6 deficiency can impair goblet cell autophagy and mucus secretion, compromising the clearance of intestinal pathogens from the mucosal surface [17]. In active ulcerative colitis (UC), the reduction in the anion exchanger of the SLC26A3 root cap membrane, essential for colonic mucin barrier formation, weakens goblet cell secretion in response to microbial challenges and decreases the core mucus component in the colonic mucus. This disruption allows bacteria to breach the inner mucus layer, reaching the epithelial cells and triggering inflammation [18]. Some microorganisms have even evolved strategies to survive within or bypass the mucosal barrier, establishing infections [19]. Currently, conventional treatment approaches often rely on symptom-driven management [20]. However, compared to solely focusing on clinical symptoms, targeting mucosal healing or inflammation control has shown to be more cost-effective in certain contexts [21].

Mucosal healing serves as a clinical marker and therapeutic target for IBD, playing a significant role as an important prognostic marker for the disease [22,23]. Ginseng, often referred to as the “king of herbs”, has garnered substantial attention for its potential disease prevention properties [24]. Ginsenosides, the primary active components of ginseng, are natural surface-active glycosides with diverse pharmacological effects [25], and their main metabolic pathways in vivo are deglycosylation and fatty acid esterification by intestinal bacteria [26,27]. Animal studies have demonstrated that saponins possess the ability to inhibit intestinal inflammation, promote intestinal barrier repair, preserve the diversity of intestinal microbiota, reduce the incidence of colon cancer in IBD, and exhibit potent anti-inflammatory effects while inducing immune homeostasis in various diseases [28,29].

Dogs are the experimental animal model used in many types of biomedical research studies [30], and some diseases in dogs are homologous to humans [31,32,33]. However, there is also growing evidence that the dog model is often not sufficiently justified and characterized as a relevant model for the human disease being studied [30]. The gut microbiota and their metabolites play a key role in the development and progression of IBD [34,35,36]. Recent studies have highlighted the similarity between canine and human intestinal microorganisms compared to other model organisms, making canine models an effective way to simulate human disease [37,38]. In this study, we used the canine model of intestinal inflammation induced by Dextran sulfate sodium (DSS) to examine the pathogenesis of canine IBD focusing on the intestinal barrier. Additionally, we evaluated the therapeutic effect of ginsenoside on canine IBD. The ultimate aim of this research is to explore a novel plant-based approach for the treatment of inflammation and provide valuable insights into canine IBD management.

## 2. Materials and Methods

### 2.1. Materials

Ginsenoside was obtained from Xi’an Season Biotechnology Co., Ltd. (Xi’an, China). Dextran sulfate sodium (DSS) salt colitis grade (used in conjunction with azoxymethane (AOM) with an average molecular weight of 36,000–50,000) was purchased from MP Biomedicals, LLC (Solon, OH, USA). Prednisone was obtained from APExBIO (Houston, TX, USA). The High Iron Diamine/Alcian Blue (HID/AB) Mucin Stain Kit (G2070) was purchased from Beijing Solarbio Science & Technology Co., Ltd. (Beijing, China). Antibodies, including Claudin-1 (1:100; 13050-1-AP), Occludin (1:200; 13409-1-AP), ZO-1 (1:100; 66452-1-Ig), and E-Cadherin (1:400; 20874-1-AP), were obtained from Proteintech (Rosemont, IL, USA).

### 2.2. Extraction and HPLC Analysis of Ginsenoside

The ginseng roots were obtained from Jilin, China. The air-dried roots were extracted and lyophilized. To begin, the dried ginseng roots were pulverized into a fine powder using a pulverizer and passed through a 40-mesh screen. Then, 100 g of the powdered ginseng sample was extracted with 70% ethanol, and the solvent of the extract solution was evaporated under vacuum. The resulting dried extract was dissolved in water and subsequently extracted with water-saturated n-butanol. The n-butanol phase was evaporated under vacuum and then lyophilized.

Before the pharmacological evaluation, the ginsenoside was analyzed using High-Performance Liquid Chromatography (HPLC). A 20 μL sample was injected into the column and eluted at room temperature with a constant flow rate of 1.0 mL/min. The mobile phase consisted of acetonitrile (solvent A) and water (solvent B). Gradient elution commenced with 17.5% solvent A and 82.5% solvent B. The elution solvents were then adjusted to 21% A for 20 min, followed by 26% A for 3 min and held for 19 min, 36% A for 13 min, 50% A for 9 min, 95% A for 2 min, and held for 3 min. Finally, the eluting solvents were returned to 17.5% A for 3 min and held for 8 min. The detection wavelength was set at 202 nm. All sample solutions were filtered through a membrane filter with a pore size of 0.2 mm. The content of the constituents was calculated using the standard curves of 32 ginsenosides. The ginsenoside content was measured twice and then averaged.

### 2.3. Animal and Treatment Protocols

All experiments were conducted on dogs approximately 3 months old. Eighteen dogs (1.90 ± 0.41 kg) were housed under controlled conditions of room temperature, humidity, and a 12/12 h light/dark cycle. The dogs were provided with two daily feedings and unrestricted access to laboratory water. The animal protocol was designed to ensure minimal pain and discomfort to the animals. The care and use of the dogs adhered to the Guidelines for the Care and Use of Laboratory Animals, as recommended by the Chinese Legislation on Laboratory Animals, Chinese Academy of Agricultural Sciences, and the Institute of Special Animal and Plant Science. Every effort was made to maximize the dogs’ welfare, minimize their pain, and minimize the total number of dogs used in the experiment. All animal procedures were approved by the Ethics Committee of the Laboratory Animal Administration of the Institute of Special Animal and Plant Sciences, Chinese Academy of Agricultural Sciences (Approval NO. ISAPSAEC-2021-60D), and strictly adhered to the animal ethics procedures and guidelines.

The experimental protocol is outlined in Supplemental Figure S1. Prior to the experiments, the dogs were acclimated to the laboratory conditions for at least 5 days. Subsequently, all animals were randomly assigned to either the control group (n = 6) or the DSS group (n = 12). To induce the IBD model, the DSS group received 2.5% DSS in place of drinking water for 5 consecutive days, while the control group (CON) was given normal drinking water [39,40]. After the administration of DSS, 6 animals were randomly selected as the model group (MOD) and anesthetized with propofol injection emulsion. Colonic mucosa and blood samples were collected under sterile conditions and stored at −80 °C, while the colon was preserved in 4% paraformaldehyde. The remaining DSS-treated animals were further divided into the GIN (ginsenoside) and PRE (prednisone) groups, which received ginsenoside and prednisone treatment, respectively. The CON group continued to be fed normally.

According to the Chinese Pharmacopoeia (2020 edition), the intragastric doses of ginsenoside and prednisone were selected as 100 mg/kg·BW/d and 2 mg/kg·BW/d, respectively, based on the conversion from the human daily dose of 6 g/60 kg body weight, using capsules [41,42,43]. The CON group received empty capsules. After 15 days of treatment (on day 20), colonic mucosa, blood, and colonic samples were obtained as described above.

### 2.4. Canine Inflammatory Bowel Disease Activity Indices

The activity of DSS-induced IBD in dogs was assessed using a numerical scoring system known as the “Canine Inflammatory Bowel Disease Activity Indices” (CIBDAIs), as previously described [44]. In summary, the CIBDAI incorporates the following parameters: attitude/activity (0 = normal, 1 = slightly decreased, 2 = moderately decreased, 3 = severely decreased), appetite (0 = normal, 1 = slightly decreased, 2 = moderately decreased, 3 = severely decreased), vomiting (0 = none, 1 = mild (once per day), 2 = moderate (two or three times per day), 3 = severe (more than three times per day)), stool consistency change (0 = normal, 1 = slightly soft feces or fecal blood/mucus, 2 = very soft feces, 3 = watery diarrhea), stool frequency (0 = normal, 1 = slightly increased (two to three times per day), 2 = moderately increased (four to five times per day), 3 = severely increased (more than five times per day)), weight loss (0 = none, 1 = mild (<5% body weight loss), 2 = moderate (5–10% body weight loss), 3 = severe (>10% body weight loss)). The scores for each parameter were summed to obtain the composite CIBDAI score, which categorizes the disease as clinically insignificant (0–3), mild (4–5), moderate (6–8), or severe (≥9) IBD. The CIBDAI scores were assessed at the same time each day, with the evaluator blinded to the treatment.

### 2.5. Histopathological Analysis

Colonic samples were fixed with paraformaldehyde and then rinsed in running water for 24 h for adequate fixation. After fixation, the samples underwent gradient ethanol dehydration. Subsequently, the samples were cleared with xylene and embedded in paraffin before being sectioned into 4 μm slices. The slides were then mounted and subjected to standard hematoxylin and eosin (H&E) staining to examine the tissue morphology. Additionally, Alcian Blue (AB) staining was performed to assess the expression of acidic mucus substances, which were identified by their deep blue coloration.

### 2.6. Immunohistochemical Analysis

Immunohistochemistry of paraffin-embedded sections was performed using the avidin–biotin indirect immunoperoxidase method. The paraffin sections were dewaxed and differentiated using a gradient ethanol series. To block endogenous peroxidase activity, a 3% H_2_O_2_ methanol solution was applied for 10 min. Next, the sections were incubated with goat serum albumin for 15 min to block non-specific binding. Subsequently, 50 μL of the primary antibody (Occludin (1:200), Claudin-1 (1:100), ZO-1 (1:100), E-Cadherin (1:400)) was added dropwise to each section. The sections were incubated at 37 °C for 2 h and then washed with PBS using gentle shaking. Following that, 50 μL of the biotinylated goat anti-rabbit IgG working solution was added dropwise to each section, followed by the addition of 50 μL of horseradish-labeled streptavidin working solution. The sections were incubated at 37 °C for an additional period. DAB chromogenic solution was used for immunohistochemical staining, followed by routine dehydration, clarification, and mounting. Positive cells were stained brown-yellow, while the nuclei were counterstained with hematoxylin to appear blue. To ensure consistent, sensitive, and reproducible staining, the slides were rocked using an antibody amplifier during the staining process.

### 2.7. Microbiota Analysis

The total genomic DNA in the samples was extracted using the SDS method. For PCR amplification, the V3-V4 region of the 16S rRNA gene was targeted using a forward primer (5′-CCTAYGGGRBGCASCAG-3′) and a reverse primer (5′-GGACTACNNGGGTATCTAAT-3′). Species annotation was performed using the QIIME 1.9.1 for 16S rRNA gene sequences, utilizing the Silva Database as the annotation database. Shannon indices and Simpson indices were calculated using the QIIME 1.9.1. The GraphPad Prism 9.4.1 was employed to analyze the differences in *α* diversity indices and bacterial composition between groups. To assess the complexity of bacterial community composition and compare differences among groups, *β* diversity was calculated based on Bray–Curtis distances using QIIME 1.9.1. Principal Coordinate Analysis (PCoA) was conducted to obtain principal coordinates and visualize sample differences in multidimensional data. The PcoA results were visualized using the ade4 and ggplot2 packages in R software (Version 2.15.3).

### 2.8. Statistical Analysis

Statistical analysis was performed using GraphPad Prism 9.4.1 software. The differences between two groups were assessed using the Mann–Whitney test during both the model and treatment phases. For the treatment phase involving three groups, the Kruskal–Wallis test was utilized. Statistical significance was defined as *p* < 0.05 and denoted by “*”, *p* < 0.01 by “**”, and *p* < 0.001 by “***”. The correlations between mucosal bacteria and physiological indices were evaluated using the Spearman rank correlation coefficient. Network visualization was conducted using Cytoscape 3.9.1 to depict the relationships between the mucosal bacteria and physiological indices.

## 3. Results

### 3.1. HPLC Analysis of Ginsenoside

A total of 28 ginsenosides were measured, and among them, 16 ginsenosides were found to have a content exceeding 1 g/kg. The chemical structures of these 16 ginsenosides are illustrated in Supplementary Figure S2A. The specific contents of the ginsenosides are presented in Supplementary Figure S2B. The contents of Rb1, Rb2, Rb3, Rc, Rd, Re, Rg1, Rg2, Rg3, Rg6, Rh1, Rh4, F1, F2, F3, and F4 in the ginseng extract were determined to be 5.28 g/kg, 7.50 g/kg, 2.75 g/kg, 7.00 g/kg, 13.9 g/kg, 15.1 g/kg, 4.57 g/kg, 7.44 g/kg, 2.73 g/kg, 4.76 g/kg, 2.39 g/kg, 9.23 g/kg, 7.54 g/kg, 3.87 g/kg, 7.71 g/kg, and 2.03 g/kg, respectively. The total ginsenoside content was calculated to be 22.27%.

### 3.2. Effect of DSS-Induced IBD in Dogs

**Figure 1:**
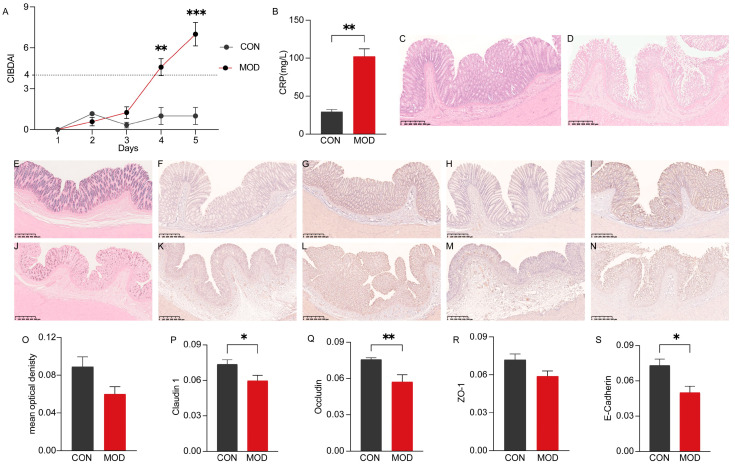
Effect of DSS-induced IBD in dogs. (**A**) Canine inflammatory bowel disease activity indices (CIBDAIs). (**B**) Comparison of C-reactive protein (CRP) levels in serum between the CON and MOD groups. (**C**) Representative hematoxylin–eosin (H.E) histological sections of the CON group. (**D**) Representative H.E histological sections of the MOD group. (**E**) Representative AB staining sections of the CON group. (**F**–**I**) Immunohistochemical staining of Claudin 1, Occludin, ZO-1, E-Cadherin in the CON group. (**J**) Representative AB staining sections of the MOD group. (**K**–**N**) Immunohistochemical staining of Claudin 1, Occludin, ZO-1, E-Cadherin in the MOD group. (**O**) Comparison of mucus expression in the colon between the CON and MOD groups. (**P**) Comparison of Claudin 1 expression in the colon between the CON and MOD groups. (**Q**) Comparison of Occludin expression in the colon between the CON and MOD groups. (**R**) Comparison of ZO-1 expression in the colon between the CON and MOD groups. (**S**) Comparison of E-Cadherin expression in the colon between the CON and MOD groups. *, ** and *** indicate *p* < 0.05, *p* < 0.01 and *p* < 0.001, respectively. CIBDAI: Canine inflammatory bowel disease activity index. CRP: C-reactive protein. H.E: Hematoxylin–eosin staining. AB: Alcian Blue staining.

To evaluate the impact of DSS-induced colitis in dogs, several parameters including CIBDAI, CRP levels, colonic sections, and related protein expression were analyzed. The results demonstrated that on the fourth and fifth days, all model animals exhibited a disease severity score (CIBDAI) of ≥4. Compared to the control group (CON), the MOD group showed significantly higher CIBDAI scores on the fifth day (*p* < 0.001) (Figure 1A). CRP, a sensitive biomarker of inflammation, was found to be significantly elevated in dogs with IBD compared to the CON group (*p* < 0.01) (Figure 1B). Histologically, the colon of the CON group displayed a normal morphology, with a fully intact mucosal surface, well-organized glands, and regular crypts (Figure 1C). In contrast, the colonic mucosal epithelium of the MOD group exhibited severe damage, including the absence of goblet cells and crypts, and the infiltration of numerous inflammatory cells in the mucosa and sublayers (Figure 1D). AB staining revealed that acidic mucus, predominantly expressed in goblet cells, exhibited a deep blue coloration. Compared to the CON group, the MOD group showed some damage to goblet cells and crypts, leading to a reduction in acidic mucus expression, although the difference was not significant (*p* > 0.05) (Figure 1O). Furthermore, the expression of tight junctions and adherens junctions was decreased to varying degrees in the MOD group. Specifically, the expression of Occludin was significantly reduced (*p* < 0.01), while Claudin 1 and E-Cadherin were significantly decreased (*p* < 0.05). The expression of ZO-1, however, did not show a significant decrease (*p* > 0.05). Representative immunohistochemical sections are shown in Figure 1.

### 3.3. Bacterial Community Analysis of Colonic Mucosa in Dogs with IBD

**Figure 2:**
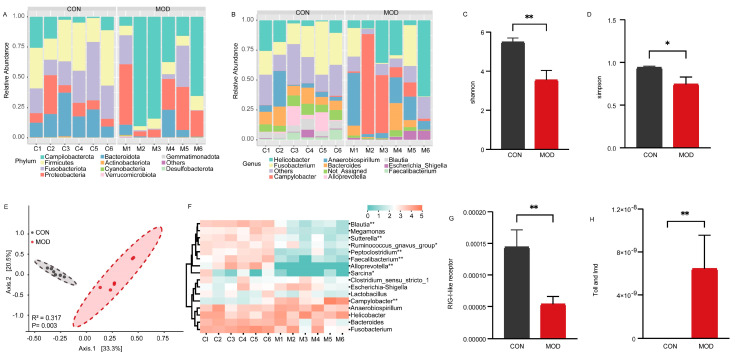
Bacterial analysis of colonic mucosa in IBD. (**A**) Bacterial composition in the colonic mucosa at the phylum level. (**B**) Bacterial composition in the colonic mucosa at the genus level. (**C**) Comparison of Shannon indices of bacteria in the colonic mucosa. (**D**) Comparison of Simpson indices of bacteria in the colonic mucosa. (**E**) Comparison of bacterial communities in the colonic mucosa. (**F**) Heatmap showing significant differences in the dominant bacterial genera of the colonic mucosa. (**G**) Comparison of RIG−I−like receptor signaling pathway of bacteria in the colonic mucosa. (**H**) Comparison of Toll and Imd signaling pathway of bacteria in the colonic mucosa. * and ** indicate *p* < 0.05 and *p* < 0.01, respectively.

The colonic mucosa comes into extensive contact with trillions of bacteria in the intestinal lumen. To investigate the influence of mucosal bacteria on the intestinal epithelial barrier, we analyzed the composition and abundance of the bacterial community. A total of 1360 amplicon sequence variants (ASVs) were obtained based on 97% sequence identity. These ASVs represented 20 phyla in the colonic mucosa of both the CON and MOD groups. At the phylum level, the most abundant bacteria in both groups were *Campilobacterota*, *Firmicutes*, *Fusobacteriota*, *Proteobacteria*, and *Bacteroidota* (Figure 2A). At the genus level, the identified ASVs were classified into 251 genera. In the CON group, the most dominant bacteria were *Fusobacterium* (24.18%), followed by *Helicobacter* (10.54%) and *Bacteroides* (10.41%), accounting for 45.13% of the overall bacterial composition. In the MOD group, the predominant genus was *Campylobacter* (26.12%), followed by *Helicobacter* (24.53%), *Anaerobiospirillum* (16.52%), and *Fusobacterium* (10.16%), accounting for 77.33% of the bacterial composition (Figure 2B). We further assessed the diversity of the bacterial community between the CON and MOD groups. Compared to the CON group, the Shannon indices were significantly decreased (*p* < 0.01) and the Simpson indices were significantly decreased (*p* < 0.05) in the MOD group (Figure 2C,D). Principal Coordinate Analysis (PcoA) based on Bray–Curtis distances revealed distinct clustering of the bacterial community composition between the CON and MOD groups, explaining at least 53.8% of the variation (*R*^2^ = 0.317, *p* = 0.003) (Figure 2E).

Furthermore, we conducted a heatmap analysis of the dominant bacterial genera with a relative abundance greater than 1% to assess their variations (Figure 2F). The results revealed notable changes in relative abundances. Specifically, the relative abundances of *Ruminococcus_gnavus_group* and *Sarcina* were significantly lower in the MOD group in comparison to the CON group (*p* < 0.05). Additionally, the relative abundances of *Blautia*, *Sutterella*, *Peptoclostridium*, *Faecalibacterium*, and *Alloprevotella* were extremely significantly lower in the MOD group compared to the CON group (*p* < 0.01). Conversely, the relative abundances of *Campylobacter* were significantly higher in the MOD group compared to the CON group (*p* < 0.01). We also investigated immunerelated differences in bacterial function and observed that the relative abundance of the RIG−I−like receptor signaling pathway was extremely significantly down-regulated in the MOD group compared to the CON group (*p* < 0.01) (Figure 2G), while the Toll and Imd signaling pathway showed an extremely significant upregulation in the MOD group compared to the CON group (*p* < 0.01) (Figure 2H).

Overall, the bacterial analysis revealed significant alterations in the composition and abundance of the colonic mucosal bacteria in dogs with IBD compared to the CON group. Moreover, the RIG−I−like receptor, Toll, and Imd signaling pathways also exhibited significant changes.

### 3.4. Ameliorative Effect of Ginsenoside on IBD in Dogs

**Figure 3:**
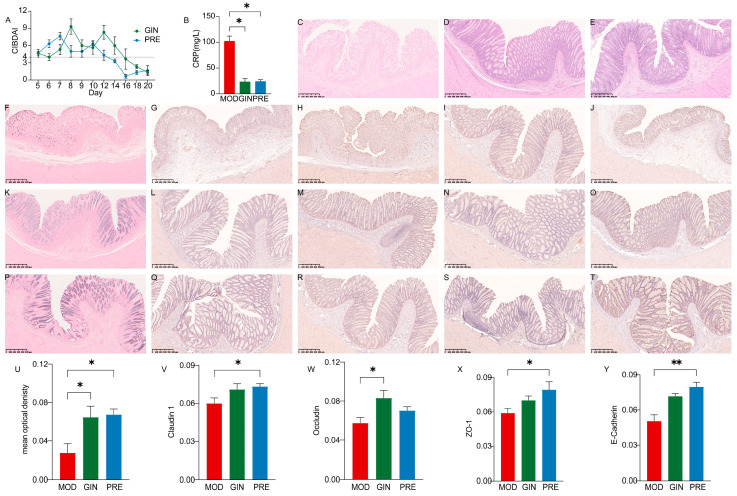
Ameliorative effect of ginsenoside on IBD in dogs. (**A**) Canine inflammatory bowel disease activity indices (CIBDAIs). (**B**) Comparison of CRP levels in serum among the three groups. (**C**) Representative H.E. staining histological sections of the MOD group. (**D**) Representative H.E. staining histological sections of the GIN group. (**E**) Representative H.E. staining histological sections of the PRE group. (**F**) Representative AB staining sections of the MOD group. (**G**–**J**) Immunohistochemical staining of Claudin 1, Occludin, ZO-1, E-Cadherin in the MOD group. (**K**) Representative AB staining sections of the GIN group. (**L**–**O**) Immunohistochemical staining of Claudin 1, Occludin, ZO-1, E-Cadherin in the GIN group. (**P**) Representative AB staining sections of the PRE group. (**Q**–**T**) Immunohistochemical staining of Claudin 1, Occludin, ZO-1, E-Cadherin in the PRE group. (**U**) Comparison of mucus expression in the colon among the three groups. (**V**) Comparison of Claudin 1 expression in the colon among the three groups. (**W**) Comparison of Occludin expression in the colon among the three groups. (**X**) Comparison of ZO-1 expression in the colon among the three groups. (**Y**) Comparison of E-Cadherin expression in the colon among the three groups. * and ** indicate *p* < 0.05 and *p* < 0.01, respectively.

To evaluate the therapeutic effects of ginsenosides on IBD in dogs, we examined the impact of ginsenoside treatment on CIBDAI, CRP levels, colonic morphology, and related protein expression. As shown in the Figure 3A, CIBDAI showed a downward trend in both GIN and PRE groups. Compared to the MOD group, both the GIN and PRE groups showed a significant decrease in CRP levels (*p* < 0.05) (Figure 3B). Histological examination with H.E. staining revealed colonic epithelial cell death, reduced goblet cell numbers, the exudation of inflammatory cells, and the partial or complete disappearance of crypts in the MOD group (Figure 3C). In contrast, the colonic structure of the GIN and PRE groups exhibited significant recovery, with a relatively intact intestinal epithelial mucosa, the gradual formation of crypt structures, and relatively complete morphological structures of goblet cells (Figure 3D,E). Additionally, compared to the MOD group, both the GIN and PRE groups demonstrated a significant increase in acidic mucus expression (*p* < 0.05) (Figure 3U). The expression of tight junctions and adherens junctions also exhibited varying degrees of improvement. Specifically, Claudin 1 and ZO-1 expressions were significantly increased in the PRE group compared to the MOD group (*p* < 0.05), while Occludin expression was significantly increased in the GIN group (*p* < 0.05), and E-Cadherin expression was extremely significantly increased in the PRE group (*p* < 0.01). Representative immunohistochemical sections are shown in Figure 3.

### 3.5. Effect of Ginsenoside on Colonic Mucosal Bacterial Community in Dogs with IBD

**Figure 4:**
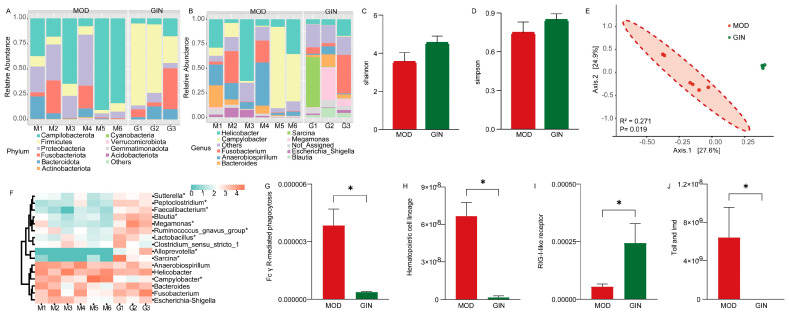
Effect of ginsenoside on colonic mucosal bacterial community in dogs with IBD. (**A**) Bacterial composition at the phylum level in the colonic mucosa. (**B**) Bacterial composition at the genus level in the colonic mucosa. (**C**) Comparison of Shannon indices of bacterial diversity in the colonic mucosa. (**D**) Comparison of Simpson indices of bacterial diversity in the colonic mucosa. (**E**) Comparison of bacterial communities in the colonic mucosa. (**F**) Heatmap showing significant differences in the dominant bacterial genera of the colonic mucosa. (**G**) Comparison of Fc γ R−mediated phagocytosis of bacteria in the colonic mucosa. (**H**) Comparison of hematopoietic cell lineage of bacteria in the colonic mucosa. (**I**) Comparison of RIG−I−like receptor signaling pathway of bacteria in the colonic mucosa. (**J**) Comparison of Toll and Imd signaling pathway of bacteria in the colonic mucosa. * indicate *p* < 0.05.

We examined the bacterial community based on 16S rRNA sequences to assess the effect of ginsenoside on colonic mucosal bacteria in dogs with IBD. The most abundant bacteria at the phylum level in the colonic mucus were *Campilobacterota*, *Firmicutes*, *Proteobacteria*, *Fusobacteriota*, and *Bacteroidota* in both the MOD and GIN groups (Figure 4A). At the genus level, *Campylobacter* (26.33%) was the most dominant bacterium in the MOD group, followed by *Helicobacter* (24.27%) and *Anaerobiospirillum* (16.19%), accounting for 66.79% of the overall bacterial composition. In the GIN group, *Sarcina* (17.43%), *Fusobacterium* (16.20%), and *Megamonas* (13.94%) were prevalent, accounting for 47.57% of the overall bacterial composition (Figure 4B). Comparing the MOD and GIN groups, the Shannon and Simpson indices, measures of bacterial diversity, showed increased values in the GIN group, although the differences were not statistically significant (*p* > 0.05) (Figure 4C,D). Principal Coordinate Analysis (PcoA) based on Bray–Curtis distance revealed significant separation between the bacterial community composition of the MOD and GIN groups (*R*^2^ = 0.271, *p* = 0.019) (Figure 4E).

Furthermore, we performed analysis on the dominant bacterial genera. The results showed that the relative abundance of *Sutterella*, *Peptoclostridium, Faecalibacterium, Blautia, Megamonas, Ruminococcus_gnavus_group, Lactobacillus, Alloprevotella,* and *Sarcina* were significantly increased in the GIN group (*p* < 0.05). Conversely, the relative abundance of *Campylobacter* was significantly decreased in the GIN group (*p* < 0.05). In terms of bacterial functional results, we observed a significant down-regulation of Fc γ R−mediated phagocytosis, the hematopoietic cell lineage, and the Toll and Imd signaling pathway in the GIN group compared to the MOD group (*p* < 0.05) (Figure 4). Additionally, the RIG−I−like receptor signaling pathway showed an extremely significant up-regulation in the GIN group compared to the MOD group (*p* < 0.05) (Figure 4I).

### 3.6. Correlation Analysis between Key Bacteria and Physiological Indices

**Figure 5:**
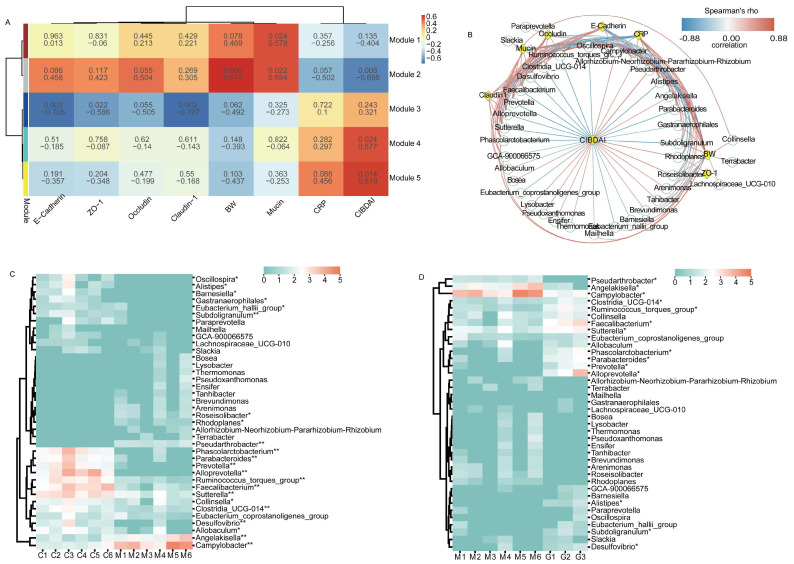
Correlation analysis between major bacteria and physiological indices. (**A**) Weighted gene co−expression network analysis. (**B**) Network plot showing Spearman’s correlation between differentially abundant bacteria and BW, CRP, and mucosal protein levels. (**C**) Heatmap showing significant differences in the modeling phase. (**D**) Heatmap showing significant differences in the treatment phase. Note: In Figure (**A**), the first row in each module represents correlation coefficients, and the second row represents *p*−values. In Figure (**B**), green circles and yellow diamonds represent significantly correlated bacteria and physiological indices (BW, CIBDAI, CRP, Mucin, Occludin, Claudin 1, and E−Cadherin), respectively. The significant correlations between bacteria and physiological indices are connected by curves, and the color of the curve lines represents the correlation strength based on the color scheme. The color scheme indicates the Spearman’s rho ranks ranging from −0.88 to 0.88. Positive and negative Spearman’s rho values represent positive and negative correlations, respectively. * and ** indicate *p* < 0.05 and *p* < 0.01, respectively.

To investigate the potential relationship between physiological indices and bacterial modules, we undertook a comprehensive analysis by clustering bacteria exhibiting similar relative abundance changes Our findings obtained 5 modules and the bacteria contained in the modules are shown in the Supplementary Table S1, a total of 37 bacteria in module 1 that were beneficial to the body and 28 bacteria in module 5 that had adverse effects on the body (Figure 5A). To explore deeper into the potential connections between the clustered bacteria and physiological indices, we conducted Spearman’s rank correlation analyses to ascertain the correlation coefficients between differentially abundant bacteria and key indicators such as body weight (BW), CRP, and mucosal protein levels (Figure 5B). Furthermore, we delved into a thorough examination of the relative abundance of these bacterial communities. Specifically, we observed a significant decrease in the relative abundance of *Oscillospira*, *Alistipes*, *Barnesiella*, *Gastranaerophilales*, *Eubacterium_hallii_group,* and *Allobaculum* in comparison to the CON group (*p* < 0.05). Moreover, the relative abundance of *Subdoligranulum*, *Phascolarctobacterium*, *Parabacteroides*, *Prevotella*, *Alloprevotella*, *Ruminococcus_torques_group*, *Faecalibacterium*, *Sutterella*, *Clostridia_UCG-014,* and *Desulfovibrio* exhibited an extremely significant decrease in comparison to the CON group (*p* < 0.01). Conversely, *Roseisolibacter*, *Rhodoplanes*, and *Collinsella* exhibited significant increments in comparison to the CON group (*p* < 0.05), and *Pseudarthrobacter*, *Angelakisella,* and *Campylobacter* demonstrated extremely significant increases in comparison to the CON group (*p* < 0.01). After ginsenoside treatment, the relative abundance of *Pseudarthrobacter*, *Angelakisella,* and *Campylobacter* exhibited a significant decrease when compared to the MOD group (*p* < 0.05). In contrast, the relative abundance of *Clostridia_UCG-014*, *Ruminococcus_torques_group*, *Faecalibacterium*, *Sutterella*, *Phascolarctobacterium*, *Parabacteroides*, *Prevotella*, *Alloprevotella*, *Alistipes*, *Subdoligranulum*, and *Desulfovibrio* displayed a significant increase in comparison to the MOD group (*p* < 0.05).

In summary, our analysis unearthed valuable insights into the intricate interplay between key bacterial genera and physiological indices. This analysis significantly contributes to our comprehension of how bacterial communities dynamically interact with and impact the physiological landscape during the progression and treatment of IBD.

## 4. Discussion

As the modeling experiments progressed, we observed several clinical manifestations in dogs with IBD, including dim hair and anal bleeding, but no significant changes in body weight (Supplementary Figure S3). Macroscopically, the inflammatory bowel segments in the abdominal cavity appeared flaccid and collapsed, with diffuse hemorrhage and edema observed on the surface of the colonic mucosa, presenting as punctate or patchy lesions (Supplementary Figure S4). Microscopically, the colonic epithelial tissue of IBD dogs exhibited severe damage, characterized by the apoptosis of colonic epithelial cells, depletion of goblet cells, and partial or complete disappearance of crypts. The colonic mucosa appeared thinner, and a significant infiltration of inflammatory cells was observed in the submucosal tissue. These findings collectively indicate the presence of a range of inflammation-related clinical symptoms, confirming the successful establishment of the DSS-induced IBD model in dogs. Following the administration of ginsenoside or prednisone, the IBD dogs showed a gradual improvement in their activity level and appetite. Notably, the colonic mucosa exhibited no apparent bleeding points, and there was a gradual restoration of crypt structure and an intact morphology of goblet cells (Supplementary Figure S4). These observations suggest that the symptoms of colonic inflammation were significantly alleviated in dogs with IBD after treatment with ginsenoside or prednisone.

The apical surface of colonic epithelial cells is protected by a two-layered mucus system. The inner layer of mucus is densely packed and firmly adheres to the epithelium, creating a barrier that prevents microbial penetration [45]. The goblet cells on the surface of the colon continuously secrete mucins, with O-linked glycans as the main component, to maintain the inner mucus layer. Meanwhile, the goblet cells in the colonic crypts secrete mucus in response to stimulation [45,46]. During the transition from the inner mucus layer to the outer layer with lower density, the numerous O-glycans on MUC2 mucin not only serve as attachment sites for microbiota but also regulate the structure, function, and transcriptional activity in the colonic mucosa [47]. This process contributes to the selective colonization of species-specific colonic microbiota [48]. In dogs, the intestinal mucus is a highly viscous and hydrated substance, with MUC2 being the most abundantly secreted component [49]. In our study, the observed decrease in the expression of acidic mucin in the colonic epithelium of dogs with IBD is likely attributed to the reduced expression of MUC2, the core component of mucin. After treatment with ginsenoside and prednisone, we observed an increase in the expression of acidic mucus in goblet cells, indicating that these treatments partially restored the properties of the mucus layer. This restoration likely prevented direct contact between the bacteria and intestinal epithelial cells, thus reducing the risk of inflammatory infections.

The tight junction proteins, including Occludin, Claudins, and Zonula occludens, are crucial for the maintenance of epithelial barrier integrity [50]. Claudin-1, for example, can induce the proliferation of colonic epithelial cells in a Notch-dependent manner [51], and increased levels of Claudin-1 may improve barrier function and reduce inflammation [52]. Occludin, a major component of tight junctions, undergoes phosphorylation/dephosphorylation processes that are crucial for tight junction regulation [53]. Elevated levels of IL-1*β* mRNA and MIR200C-3p in intestinal tissues can lead to decreased Occludin expression, thereby increasing tight junction permeability [54]. ZO-1 is critical for epithelial repair and contributes to Wnt-β-catenin signaling and mitotic spindle orientation [55]. The down-regulation of ZO-1 in experimental IBD compromises mucosal repair and promotes disease progression. E-Cadherin, an integral component of adherens junctions, is essential for cell adhesion and maintaining the epithelial phenotype. Loss of E-Cadherin expression results in the loss of contact inhibition and is associated with increased cell motility and advanced stages of cancer [56]. Tight junctions and adherens junctions serve as important indicators for evaluating intestinal barrier function. Our findings demonstrate disrupted colonic tissue structure in dogs with IBD, accompanied by varying degrees of down-regulation in the expression of tight junctions and adherens junctions. This disruption compromises the integrity and function of the colonic epithelial barrier. Treatment with ginsenoside leads to an increased expression of Occludin, and prednisone leads to an increased expression of Claudin-1, ZO-1, and E-Cadherin. These changes contribute to the restoration of intestinal structure and intestinal barrier permeability, inhibiting the invasion of microbial antigens from the intestinal lumen, blocking the continuous antigen stimulation of enterocytes, and alleviating inflammatory symptoms.

Increased intestinal permeability has been identified as a precursor to the onset of IBD, and this heightened permeability is attributed to a dysregulated immune response against the normal gut microbiota [57]. The colonic mucosa, with its extensive surface area, is in direct contact with trillions of microorganisms that colonize the intestinal lumen [58]. Compared to fecal samples, mucosal samples provide a more accurate representation of potential microbial dysbiosis in IBD [42]. However, limited information is available regarding the mucosal microbiota in dogs with IBD at the time of diagnosis and in response to medical treatment. In our study, we observed reduced bacterial diversity in the colonic mucosa of dogs with IBD, with *Campylobacter* as the dominant bacteria, followed by *Helicobacter*, *Anaerobiospirillum*, and *Fusobacterium*. Moreover, we observed a significant decrease in the relative abundance of *Firmicutes* and a non-significant increase in *Proteobacteria* in dogs with IBD, consistent with findings from other IBD studies [59,60]. Previous research has reported a higher abundance of *Proteobacteria* and lower abundance of *Bacteroides*, *Eubacterium,* and *Faecalibacterium* in IBD patients compared to healthy individuals [61,62]. In our study, we also found a decreased relative abundance of *Bacteroides* and *Fusobacterium*, while *Anaerobiospirillum* and *Helicobacter* showed an increased abundance to some extent. Limited studies have reported on *Anaerobiospirillum*, which has only been isolated from the feces of humans, dogs, and cats with diarrhea, as well as patients with bacteremia [63,64,65,66,67]. It has also been found in cats with ileocolitis [68], exhibiting morphological and characteristic similarities to *Campylobacter* [69]. *Helicobacter pylori* (*H. pylori*) infection has been implicated in the pathogenesis of IBD through its potential to induce changes in gastrointestinal permeability or to trigger immune disorders via various immune pathways, resulting in antigen absorption and autoimmunity. However, epidemiological data suggest a low prevalence of *H. pylori* infection in IBD patients, and this infection may have a protective role in IBD development. The relationship between *H. pylori* and IBD remains unclear and requires further investigation [70,71,72,73]. In our study, we observed an increased relative abundance of *Helicobacter* in the colonic mucosa of dogs with IBD, suggesting its potential involvement in IBD pathogenesis.

Treatment with ginsenoside led to a partial restoration of mucosal bacterial diversity in dogs with IBD. The relative abundance of *Campylobacter*, the primary bacteria implicated in IBD, was significantly decreased. It has been demonstrated that *Campylobacter jejuni* can compromise the integrity of the intestinal mucosal barrier, facilitating its own transmission and promoting the translocation of non-invasive microbiota across the intestinal epithelium through epithelial lipid rafts of M cells [74,75]. Such an invasion of microorganisms can disrupt the intestinal epithelial barrier [76], potentially triggering inflammatory responses in susceptible individuals [77]. Additionally, bacteria that exhibited decreased abundance in IBD dogs, including *Sutterella*, *Sarcina*, *Faecalibacterium*, *Alloprevotella*, *Blautia*, *Peptoclostridium,* and *Ruminococcus_gnavus_group*, showed increased abundance following ginsenoside treatment.

Although the exact pathogenesis of IBD remains unclear, studies have suggested that alterations in the interaction between intestinal microbes and the mucosal immune system in susceptible individuals contribute to intestinal inflammation [78,79]. In dogs with specific IBD conditions, such as Yorkshire Terrier enteropathy and chronic canine IBD, reductions in the relative abundance of *Alloprevotella*, *Phascolarctobacterium*, *Prevotellaceae*, *Oscillospirales*, *Oscillospiraceae UCG-005*, *Phascolarctobacterium*, *Succinivibrionaceae*, and *Succinivibrio* have been observed [80,81]. Notably, the decreased abundance of *Phascolarctobacterium* has been associated with colon inflammation, regardless of the specific IBD phenotype [82]. Our results show that there is a certain correlation between the bacterial community of the colonic mucosa and the physiological indices. We analyzed the correlation between 65 kinds of clustering bacteria and physiological indices, and a total of 38 kinds of bacteria were correlated with physiological indices. Among them, *Clostridia_UCG-014*, *Ruminococcus_torques_group*, *Faecalibacterium*, *Sutterella*, *Phascolarctobacterium*, *Clostridia_UCG-014*, *Ruminococcus_Torques_group*, *Faecalibacterium*, *Sutterella*, *Phascolarctobacterium Parabacteroides*, *Prevotella*, *Alloprevotella*, *Alistipes*, *Subdoligranulum,* and *Desulfovibrio* were significantly decreased in IBD, and *Pseudarthrobacter*, *Angelakisella,* and *Campylobacter* were significantly increased. All were significantly altered after ginsenoside treatment. Indeed, it is widely recognized that many animal disease models exhibit a self-healing phenomenon. Notably, there have been reports indicating that DSS-induced IBD demonstrates spontaneous recovery. This recovery becomes evident not only through the natural restoration of physiological indices and inflammatory cytokines (such as IL-17A, IL-6, and CRP), but also through the inherent capacity of intestinal microorganisms to self-regulate and recover (the abundances of *Lactobacillus* and *Alistipes* tend to decrease, while those of *Streptococcus*, *Escherichia–Shigella*, and *Oscillibacter* tend to increase) [83]. However, our research findings reveal significant shifts in specific dominant bacterial community. Specifically, *Campylobacter*, *Alloprevotella*, *Sutterella*, and *Faecalibacterium* exhibited a significant decrease during the progression of IBD, and their abundance significantly increased following treatment with ginsenoside. Furthermore, our study identified a correlation between the presence of these bacteria and the physiological indices of the animals. These observations underscore the distinctive role of these bacterial taxa in the context of IBD and their responsiveness to ginsenoside treatment. This finding is noteworthy because it indicates that the changes in the gut microbiota treated by ginsenoside treatment are distinct from the natural changes that occur during the self-healing process. These specific alterations in the microbiota composition may play a role in the therapeutic effects against IBD by ginsenoside.

## 5. Conclusions

In conclusion, our study provided insights into the pathogenesis of DSS-induced canine IBD, focusing on histopathological changes and mucosal microbiota alterations. The results highlight that the destruction of tight junctions and adherens junctions in the colonic epithelium contributes to the pathological symptoms observed in IBD. Furthermore, the crucial role of mucus layer properties and its core components is in maintaining the integrity of the mucosal barrier and preventing bacterial infiltration and subsequent inflammation. Ginsenoside treatment demonstrated its effectiveness in regulating the relative abundance of some bacteria in the colonic mucosa, especially dominant bacteria such as *Campylobacter*, *Alloprevotella*, *Sutterella,* and *Faecalibacterium*. This modulation positively influenced the construction of the intercellular protein network, mucin secretion, and the overall integrity of the colonic epithelial barrier, thereby promoting the recovery of IBD.

## Data Availability

The data that support the findings of this study are available from the corresponding author, [C.X.], upon reasonable request.

## Supplementary Materials

The following supporting information can be downloaded at https://www.mdpi.com/2076-2607/11/11/2616/s1, Figure S1: Experimental protocol. Figure S2: Chemical structures and HPLC analysis of 16 major ginsenosides. (A) The chemical structures of determined ginsenosides(B) The contents of ginsenosides. Figure S3: Comparison of body weight among the groups. Figure S4: Analysis of colonic mucosal barrier integrity in dogs with IBD; Table S1: Bacterial module.
