# Correction: Mao et al. Gut Bacterial Community Determines the Therapeutic Effect of Ginsenoside on Canine Inflammatory Bowel Disease by Modulating the Colonic Mucosal Barrier. *Microorganisms* 2023, *11*, 2616

**DOI:** 10.3390/microorganisms12010158

**Published:** 2024-01-12

**Authors:** Aipeng Mao, Weigang Zhao, Yuhang Zhu, Fantao Kong, Danyang Chen, Huazhe Si, Chao Xu

**Affiliations:** 1 Institute of Special Animal and Plant Sciences, Chinese Academy of Agricultural Sciences, Research Center for Microbial Feed Engineering of Special Animals in Jilin Province, Innovation Center for Feeding and Utilization of Special Animals in Jilin Province, Changchun 130112, China; mao443418199@163.com (A.M.); zwg1163@126.com (W.Z.); kongfantao@caas.cn (F.K.); chendanyang@caas.cn (D.C.); 2 College of Animal Science and Technology, Jilin Agricultural University, Changchun 130118, China; yuhangzhu199512@163.com

The authors wish to make the following corrections to this paper [1]:

In Section 1: Introduction, paragraph 4, lines 1–2, the author mistakenly wrote: “Dogs are excellent experimental animal models in many types of biomedical research studies, and most diseases in dogs are homologous to humans [30–33]”.

The correct version should be as follows:

“Dogs are the experimental animal model used in many types of biomedical research studies [30], and some diseases in dogs are homologous to humans [31–33]. However, there is also growing evidence that the dog model is often not sufficiently justified and characterized as a relevant model for the human disease being studied [30].”

The reference order will also be updated accordingly.

The authors apologize for any inconvenience caused and state that the scientific conclusions are unaffected. The original publication has also been updated.
