# Supplementary material for: Gut Bacterial Community Determines the Therapeutic Effect of Ginsenoside on Canine Inflammatory Bowel Disease by Modulating the Colonic Mucosal Barrier
Source: Microorganisms. 2023 Oct 24;11(11):2616. doi: 10.3390/microorganisms11112616 (PMC10672857; doi:10.3390/microorganisms11112616)
Supplement: Supplementary file 1 [file microorganisms-11-02616-s001.zip › Supplemental files.pdf]

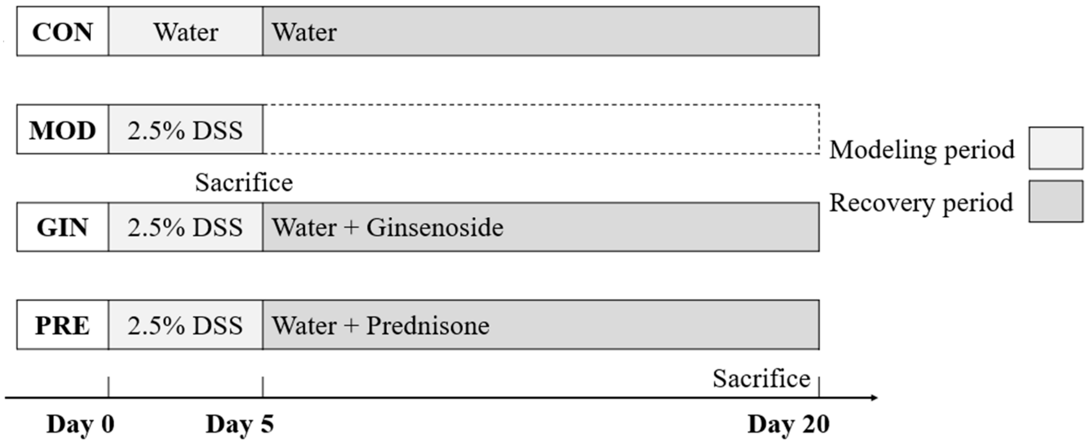

Supplemental Figure S1 Experimental protocol. Experimental dogs were divided into four groups, i.e., control group (CON), model group (MOD), ginsenoside group (GIN), prednisone group (PRE). Animals in the model, ginsenoside and prednisone group received 2.5% (DSS) in drinking water for 5 consecutive days. After the DSS administration, dogs in GIN received oral ginsenoside 100mg/kg while in PRE also received prednisone 2mg/kg for 15 consecutive days (Day 1-5 is the modeling period and then up to 20 days is the recovery period).

A

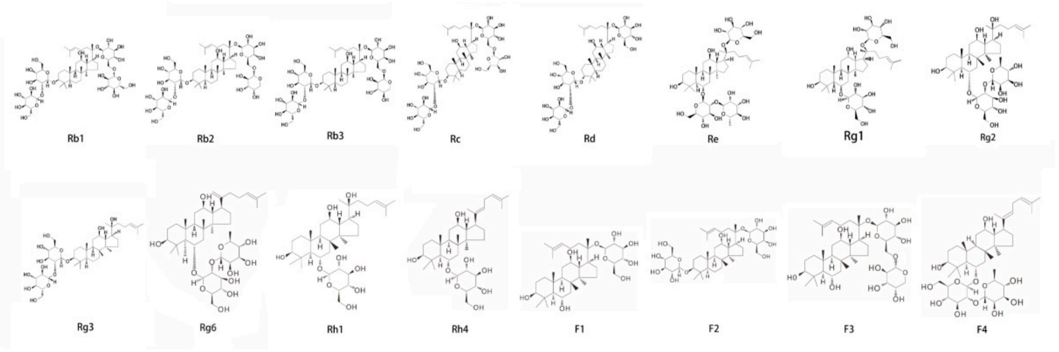

B

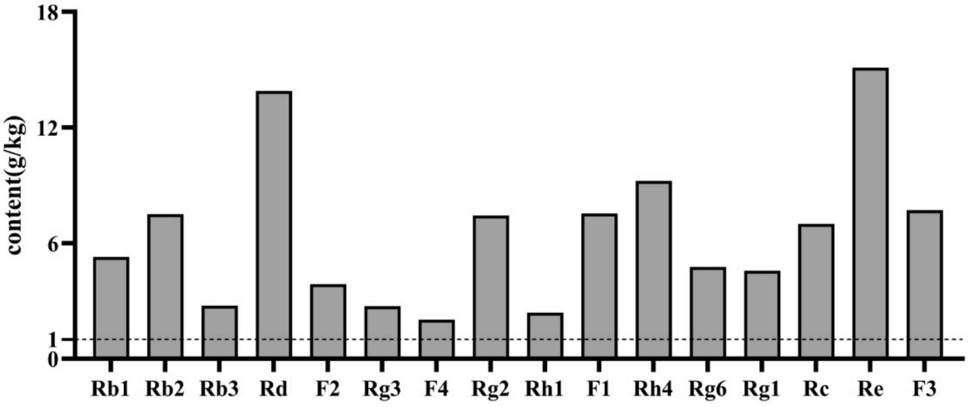

Supplemental Figure S2. Chemical structures and HPLC analysis of 16 major ginsenosides. The chemical structures of determined ginsenosides are shown in A. The contents of ginsenosides are shown in B.

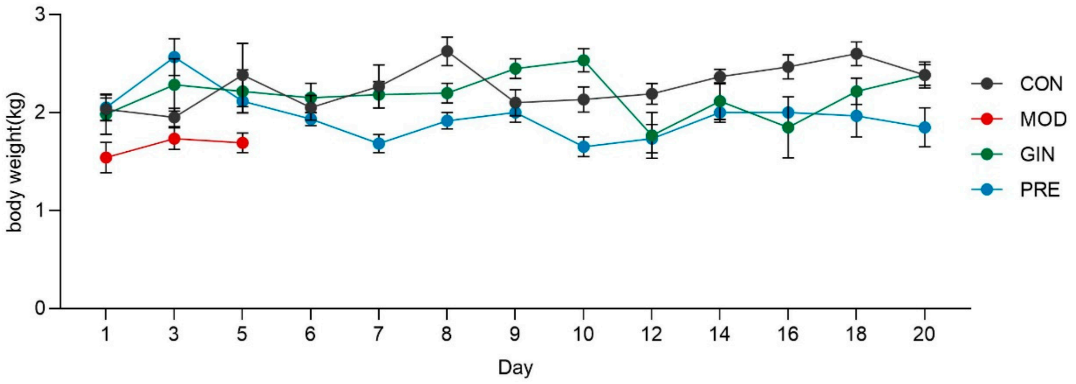

Supplemental Figure S3. Comparison of body weight among the groups.

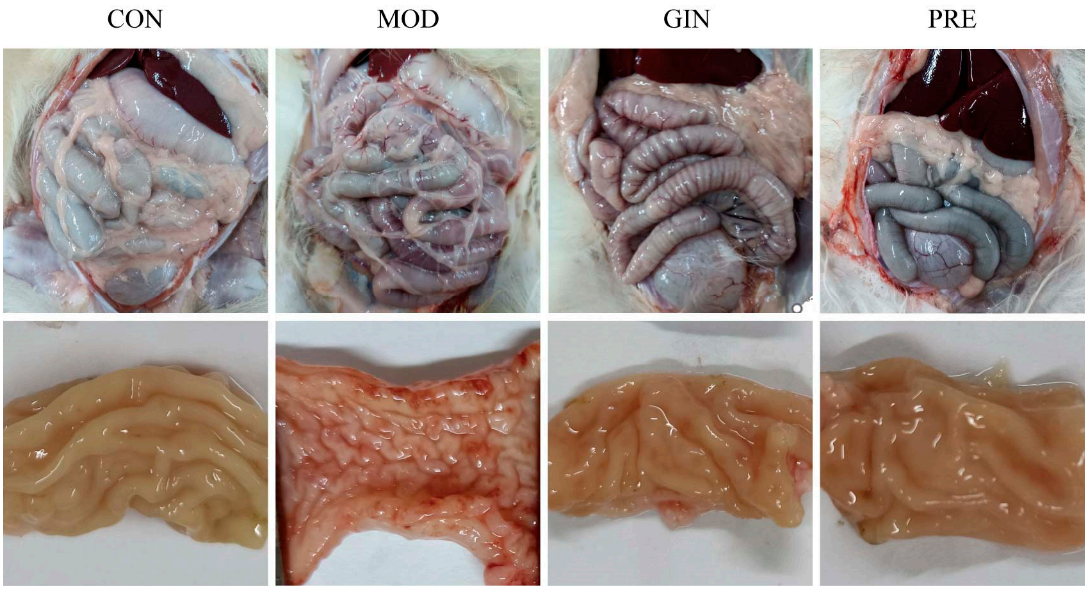

Supplemental Figure S4. Analysis of colonic mucosal barrier integrity in dogs with IBD. In normal dogs, the inner surface of the colon is smooth and the inner wall is everted, in the IBD dogs, there were intestinal inflammatory lesions extensively throughout the colon, and the mucosal surface of the colon showed obvious symptoms of plaque bleeding, deep red bleeding symptoms were observed in the abdominal gut wall, and diffuse hemorrhage and edema were observed in the inner wall of the colon. However, the abdominal symptoms of GIN and PRE groups were mild, and there was no obvious bleeding site in the colon wall. The above results indicate that bloody diarrhea gradually disappeared and activity gradually resumed after treatment with ginsenoside or prednisone, and the symptoms of IBD in dogs were relieved to some extent.
